# 

**Published:** 1881-06

**Authors:** 


### THE ELEVENTH ANNUAL MEETING OF THE WISCONSIN STATE DENTAL SOCIETY.

Will be held in Milwaukee, on the third Tuesday in July, 1881, and continue three days. It is hoped that every member of the society, and dentists from every part of the State will attend this session, and contribute to its interest and success. Don't neglect or postpone this duty.

### SUBJECTS.

#### Conservative and Operative.

1. Morbid conditions of the Gums and Treatment.
2. Diseases of the Associate parts of the mouth and their Treatment.
3. Anomalous Cases and incidents in office practice.

MECHANICAL.

1. The relative merits of different materials as a base for artificial teeth.

2. Pivot-teeth, in what cases and in what manner can they be successfully inserted.

3. The effectiveness of different appliances for regulating teeth.

ORAL CHEMISTRY AND THERAPY.

1. The benefits and abuses of Amalgam and Cements—Compatibility of filling materials with tooth bone.

2. What is the best pain obtunder for sensitive dentine—new dental remedies.

3. Do poisonous articles extensively used in adulterating food register themselves upon the teeth?

Give these subjects careful thought, and personally assist the committee to make this session the most interesting and profitable one ever held. Complete announcement will be issued in due time by the Executive Committee:

EDGAR PALMER,
R. G. RICHTER,
W. F. LEWIS.

### CHANGE OF TIME.

The Annual Meeting of the American Dental Association will take place this year on Tuesday, the 12th of July, instead of the first Tuesday of August, as heretofore, and as was expected, till within a recent period.

This change is rendered necessary from the fact that quite a large number of the active members will not be able to attend at the usual time. It is very desirable that there be a full attendance of the members, and of the profession generally.

---

### THE KENTUCKY STATE DENTAL SOCIETY.

The eleventh Annual Meeting of the Kentucky State Dental Society will be held in the city of Lexington on Tuesday, June the 7th. The programme is as follows:

| | |
|---|---|
| ANATOMY . . . . . . . . . . . . | W. Van Antwerp. |
| PHYSIOLOGY . . . . . . . . . . . | A. W. Smith. |
| HISTOLOGY . . . . . . . . . | |
| PATHOLOGY . . . . . . . . . . . | A. O. Rawls. |
| THERAPEUTICS . . . . . . . . . | F. Peabody. |
| OPERATIVE DENTISTRY . . . . | J. Hooper, J. B. Kidd. |
| MECHANICAL " . . . . | J. F. Canim, E. W. Ruth. |

Let the profession in Kentucky see to it that this meeting is just as good as they can make it; and, if this is done, it will bea success. A cordial invitation and welcome is extended to all.

### SPECIAL NOTICE.

### American Dental Association.

The next Annual Meeting of this Association will be held in the City of New York,

ON TUESDAY, JULY 12, 1881.

This change of time has been decided upon by the Executive Committee and the officers of the Association, in view of the fact that some forty or fifty of the members will probably be absent at the international congress, to be held in London on the 2d–9th August.

Circulars will be sent to all members of the Association, with further particulars.

C. N. Peirce, *Pres't.*

Geo. H. Cushing, *Sec.*

J. N. Crouse, *Chairman.*

W. H. Morgan,

G. V. Black,

Geo. W. Keely,

Chas. D. Cook,

C. S. Stockton,

C. N. Peirce,

S. G. Perry.

*Executive Committee.*

### SOUTHERN DENTAL ASSOCIATION.

The thirteenth Annual Meeting of this Association will take place at Ashville, North Carolina, on the last Tuesday in July (26th), 1881.

The North Carolina Dental Association meets at the same time and place.

Every arrangement that will conduce to the pleasure of the occasion has and will be made.

All Dentists are invited to be present at this delightful place, and have a good time.

E. S. Chisholm, *Rec. Sec. S. D. A.*

### ALABAMA DENTAL ASSOCIATION.

The next Annual Meeting of the Alabama Dental Association will be held in Selma, Ala., on the third Tuesday (19th day) of July, 1881.

The State Board of Dental Examiners will meet at same time and place. Every Dentist practicing in the State is expected to be present, as under the late act of the legislature, every one practicing dentistry in the State is *compelled to have* a license from this Board.

T. M. Allen, *Recording Secretary, Eufaula, Ala.*

### TO THE AMERICAN DENTAL ASSOCIATION.

Arrangements have been made for round trip fare from Cincinnati to New York for those going to the American Dental Association to be held on the 12th of July, at greatly reduced rates, over one of the most pleasant routes. For particulars and tickets apply to the Editor of the Dental Register, Cincinnati. All delegates passing through this city can avail themselves of this arrangement.

### CONTENTS.

### COMMUNICATIONS.

### THE DENTAL REGISTER,

A MONTHLY MAGAZINE DEVOTED TO THE INTERESTS OF

**THE DENTAL PROFESSION.**

*TERMS REDUCED TO $2.50 PER YEAR. SINGLE COPIES 25C.*

EDITED BY PROF. J. TAFT, D.D.S.

AND

*Published by SPENCER, CROCKER & CO., Ohio Dental Depot.*

The volume commences in January and closes in December.

The REGISTER being issued monthly is always fresh, therefore Indispensable to the practitioner who desires to keep posted up with the new inventions and improvements which are daily developed. Neither expense nor effort will be spared to give value and variety to its contents. Articles will be illustrated with well executed engravings whenever they will add to the interest or assist to explanation.

. Its extensive circulation and frequency of issue make it one of the BEST DENTAL ADVERTISING MEDIUMS in the United States.

All subscriptions must begin with January or July.

Local or special notices, five lines or less, will be inserted for$3 each insertion; over five lines, 50 cts. per line.

All advertising bills payable in advance, or at furthest, after the issue of the first number containing the advertisement. All transient advertisements to be paid for in advance.

☞CONTRIBUTIONS SOLICITED.

Exclusively Editorial Communications should be addressed to Editor.

The latest number of the REGISTER may be ordered with or without oth-☞ goods at any time, and all letters should be addressed to

*SPENCER, CROCKER & CO., Ohio Dental Depot, Cincinnati, O.*

